# Breast and Nipple Dermatoses During Lactation

**DOI:** 10.1111/ajd.14586

**Published:** 2025-08-15

**Authors:** Hamish Moore, Annabel Stevenson

**Affiliations:** ^1^ Faculty of Health and Medical Sciences University of Adelaide Adelaide South Australia Australia; ^2^ Department of Dermatology Royal Adelaide Hospital Adelaide South Australia Australia; ^3^ Department of Dermatology Queen Elizabeth Hospital Woodville South South Australia Australia

**Keywords:** breast feeding, dermatitis, eczema, lactation, mastitis, medication safety, psoriasis

## Abstract

Lactation and breastfeeding can present both psychological and physical challenges for breastfeeding mothers. In addition, many nursing mothers will also suffer from breast and nipple dermatoses during this period, compounding these difficulties. Common causes of breast and nipple dermatitis during lactation include eczema, psoriasis, mastitis, mammary Paget's disease, Raynaud's phenomenon, and herpes virus infection, all of which may arise or be exacerbated during breastfeeding. This article summarises the common causes of breast and nipple dermatitis during lactation, as well as their investigation and management. In addition, we review the safety of common dermatological medications in this population, accounting for the unique consideration of the breastfeeding infant.

## 1 Introduction

Breastfeeding is a method of infant nutrition that provides numerous benefits for both newborns and their mothers. Both the World Health Organisation (WHO) and the Australian National Health and Medical Research Council (NHMRC) recommend exclusive breastfeeding for the first six months of an infant's life, followed by the introduction of complementary foods in conjunction with breastfeeding for up to two years or beyond. Benefits include the facilitation of growth, development, and excellent long‐term health outcomes for infants, while also nurturing the bond between mother and child.

Lactation can present both psychological and physical challenges for breastfeeding mothers. Breastfeeding demands a significant time commitment, often leading to exhaustion and sleep deprivation, and similarly competes with logistical hurdles such as returning to work. In some mothers, feeding can cause trauma and pain, resulting in feelings of frustration and low self‐esteem.

A substantial number of patients also suffer from dermatoses of their breasts or nipples whilst breastfeeding. Breast and nipple dermatoses can manifest in different forms, some of which specifically arise and some of which are exacerbated during lactation. These conditions may cause discomfort and pain; eventually leading to a cessation of breastfeeding.

Some women may also be lactating for long periods whilst requiring treatment for other dermatological conditions. Doctors should be aware of the safety of potential treatments (topical, physical, and systemic) for the breastfeeding infant of a mother being treated with these modalities, acknowledging that trials often exclude lactating women.

This article presents a scoping review of breast and nipple dermatoses during lactation and breastfeeding, including synthesised guidelines on the medicines used to treat them.

## 2 Breast Dermatoses in the Lactating Woman

### 2.1 Inflammatory Dermatoses

#### 2.1.1 Eczema

Eczematous dermatoses of the nipples, including endogenous atopic dermatitis, irritant contact dermatitis, and allergic contact dermatitis, are at risk of development or exacerbation during lactation. Eczema presents as an erythematous eruption of the affected area. In acute cases, this is associated with oozing, crusting, and erosions of the skin, and subacutely, scaling or lichenification is seen. Symptomatically, pain and itch are common features [1, 2, 3]. Isolated eczema of the nipple and areola can occur in all patients suffering from eczema [2], though several additional factors predispose patients to the condition during the lactation period, and ultimately may hinder the breastfeeding experience.

##### 2.1.1.1 Irritant Contact Dermatitis

**FIGURE 1:**
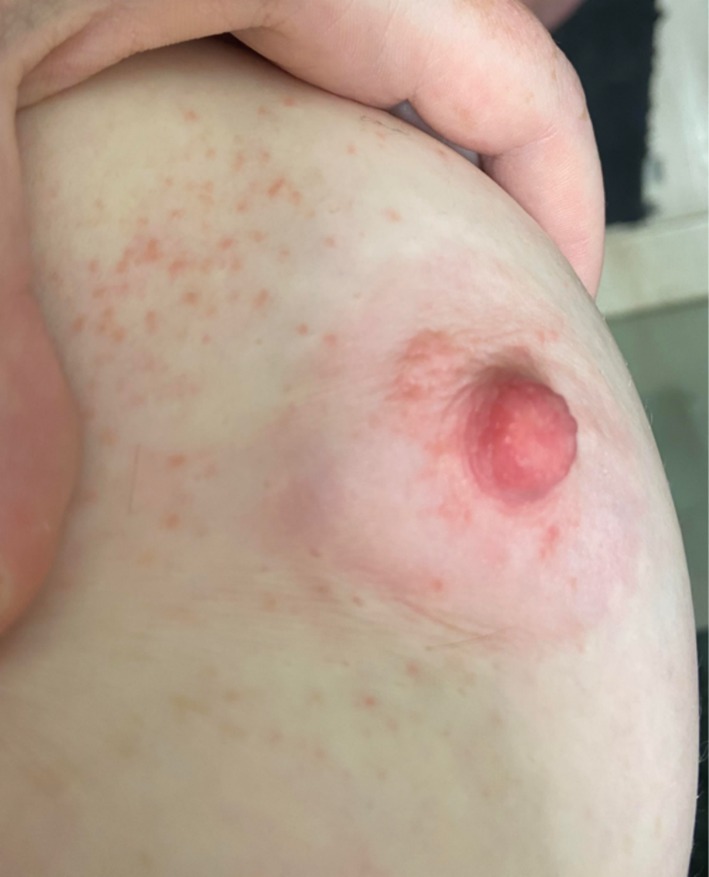
Irritant contact dermatitis of the nipple. Image provided by Dr Annabel Stevenson.

Irritant contact dermatitis (ICD) is caused by direct physical or chemical trauma to the affected skin. In relation to the breasts and breastfeeding, the areola is most affected, with the nipple and the skin immediately adjacent typically, though not always, spared, as seen in Figure 1.

The most common trigger is trauma from the feeding newborn. Poor latch from the newborn results in repetitive stretching of desmosomes in the areolar skin. This results in microhaemorrhage and release of histamines and inflammatory cytokines, leading to the development of dermatitis [4]. Trauma from breastfeeding is compounded in cases of infantile congenital abnormalities, such as cleft lip and palate, or the use of an ill‐fitting breast pump. Owing to the changes in shape of the breast and nipples during lactation, frictional trauma from ill‐fitting bras and clothing can also occur.

Mechanical skin damage may be exacerbated by overexposure to moisture and maceration of the nipple–areolar complex [4]. Prolonged and recurrent skin exposure to infant saliva results in skin inflammation and erosion termed moisture‐associated skin damage (MASD). Additional exposure to the acidity and viscosity of saliva and salivary enzymes also contributes to MASD, which clinically presents as irritant contact dermatitis [5]. Low oestrogen levels during lactation may additionally lead to menopause‐like “hot flushes” and xerotic skin, causing increased sweating and subsequent MASD, as is seen in jogger's nipples. Breast milk, itself, may contribute to an increase in moisture, but unlike saliva or other bodily fluids, this contains antibodies, epidermal growth factor, and erythropoietin, and its application has been shown to reduce inflammation in many cases [6]. Occlusion from padded bras and nipple covers, as well as the application of topical medications and emollients, can also contribute to moisture excess [4].

Exploration of personal hygiene practices may reveal factors leading to chemical irritation. Soaps and detergents have been reported amongst the commonest causes of ICD in Australia, and an increase or change in their use may be present post‐partum. Laundry products may additionally be retained in clothing or bras. Whilst there have been no cases specifically reported in a lactating mother, granular parakeratosis may result from the combination of increased friction to the breast region and exposure to personal care products containing benzalkonium chloride. This occurs through disruption of cellular lipid membranes and inactivation of skin enzymes, and frequently affects the inframammary folds [7].

##### 2.1.1.2 Allergic Contact Dermatitis

Allergic contact dermatitis (ACD), a type IV hypersensitivity reaction, develops following repeat exposure to an allergen. The risk of developing ACD of the nipple or breast is at its highest during the postnatal period. ACD occurs at the site of allergen contact, and so in comparison to ICD, allergic dermatitis is more likely to affect the nipple itself.

As with ICD, the patient's hygiene practices are an important risk factor. Soaps, detergents, shampoo and conditioner, fabric softeners, and textiles may contain potential allergens, and again the possibility of products being retained in clothing is noted. The fragrances, preservatives and other ingredients used to produce such products make up some of the commonest allergens in Australia, and with changes in behaviour over the lactation period, the nursing mother is at risk of new exposure. For example, a case of ACD from methylisothiazolinone contained in baby wipes has specifically been reported in Australia [8] and contact allergy to propylene glycol used in bra padding [9], and disperse blue 106 fabric dye [10] have also been reported in cases affecting the breasts.

During breastfeeding, the use of nursing adjuncts such as nipple shields, protection covers and shells has become increasingly popular. These products are typically made of silicone, plastic, rubber, or silver. Whilst silicone and plastic are uncommon causes of ACD, contact allergy to silver is consistently reported in the literature [11]. Accelerators (thiurams, carbamates, thiazoles, and thioureas) and antioxidants (mainly p‐phenylenediamine (PPD) derivatives) used in rubber and latex production are also common allergens [12]. Nickel and cobalt, often used as the base of silver‐plated products, including nipple shields, are two of the most frequent causes of ACD, and may also be found in the underwire of bras [13, 14]. In a reported case, one 17‐year‐old girl presented with ACD on her breast and was found to have a contact allergy to the nickel in the mobile phone that she carried in her bra [15]. In breastfeeding infants who are concurrently consuming solid food, especially in cases where the introduction of food coincides with the development of nipple dermatitis, allergy to residual food in the infant's mouth is a theoretical, though unreported, cause. ACD to infant formula has been reported in one case of an adoptive mother using a supplemental nursing system [16].

The topical treatments used to manage breast and nipple discomfort during lactation and breastfeeding are numerous and varied, with many of these a potential cause of ACD. Home remedies might include the application of beeswax [17], chamomile [18], garlic [19] and aloe vera [20], all of which have been reported allergens [2]. Emollients containing vitamins A and E [21], as well as various preservatives or fragrances have equally been implicated. Lanolin, a purified wax composed using the wool of sheep, is frequently used to soothe painful nipples during breastfeeding. Again, this may lead to ACD and should be avoided in patients with a known sheep or wool allergy [22]. Prescribed topical medications equally pose a risk. Topical corticosteroids, antibiotics (e.g., neomycin or bacitracin), and topical anaesthetics (e.g., benzocaine) are commonly reported causes of contact allergy. Miconazole ointment, which is often used during lactation, and Trimovate cream have been reported in cases affecting the nipple and breasts respectively [23, 24]. Patch testing should target not only the active ingredient, but include any excipients, such as in the latter case, where the patient reacted to cetearyl alcohol, sodium metabisulfite and clobetasone butyrate upon patch testing [24].

Allergens reported to have caused breast and nipple ACD are summarised in Table 2.

##### 2.1.1.3 Atopic Dermatitis

**FIGURE 2:**
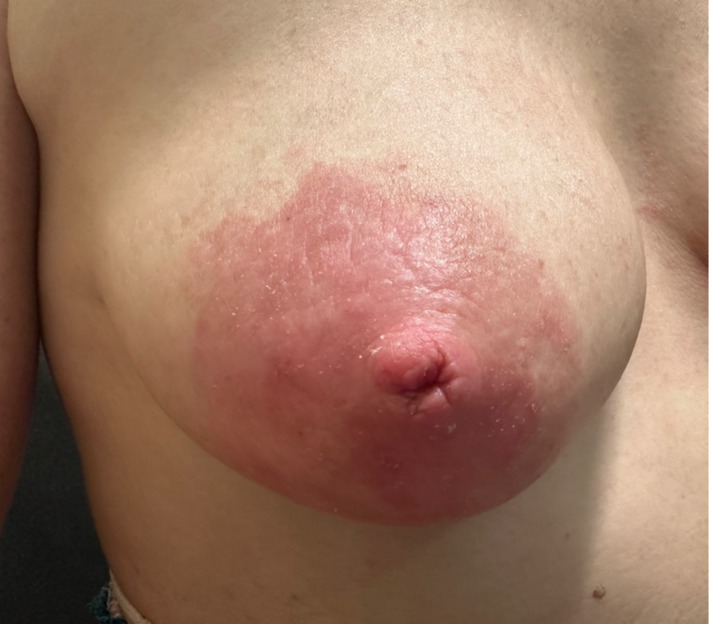
Atopic dermatitis of the nipple. Image provided by Dr Annabel Stevenson.

Whilst more common in children, 5%–10% of adult Australians suffer from atopic dermatitis [25]. The nipples are commonly affected (Figure 2), with one study in paediatric patients demonstrating involvement in up to 23% of patients [26]. The condition results from a complex interplay of skin barrier dysfunction, immune dysregulation, and cutaneous microbiome imbalance [25].

Similar to other eczematous dermatoses, breastfeeding, through mechanical irritation and exposure to moisture and irritant chemicals, can lead to exacerbation of nipple eczema during the post‐partum period [2]. Atopic patients also frequently suffer from allergic contact dermatitis because of their chronic skin breakdown and exposure to antigens [1, 25]. Around half of the women who suffer from breast dermatoses during lactation are believed to be atopic [2, 3].

#### 2.1.2 Psoriasis

Psoriasis of the nipple or breast is uncommon but is an important differential diagnosis. The condition has been described as having a bimodal age of onset, with the first of these (20‐30 years) coinciding with childbearing age. Between 40%–90% of women with psoriasis suffer an exacerbation of their disease during the post‐partum period [27, 28, 29].

Psoriasis of the nipple or breasts presents as erythematous plaques with overlying scale, usually more well‐demarcated than eczema. The “classically” described Koebner Phenomenon is particularly noteworthy in breastfeeding mothers, with latch from the nursing infant causing trauma and potential exacerbations of the disease [1, 28]. The condition itself is not a contraindication to breastfeeding; though the discomfort and exacerbations of disease in this population are often cited as reasons for discontinuing breastfeeding.

### 2.2 Malignancy

#### 2.2.1 Mammary Paget's Disease

Mammary Page's disease represents 1%–3% of all breast cancers and is an important differential diagnosis for inflammatory breast dermatoses. This rare form of intraductal carcinoma invades the epithelium of the nipple, spreading to the adjacent areola, and presents as scaling, crusting, and erythema of the unilateral breast. Whilst Mammary Page's Disease most commonly affects post‐menopausal women [3, 30], it has been reported in a 32‐year‐old lactating mother who had her symptoms dismissed as lactation‐associated changes for one year before her ultimately fatal diagnosis [31]. Suspicions should be raised in patients with unilateral breast or nipple changes, and those with symptoms persisting longer than three weeks [3, 30].

### 2.3 Infections

#### 2.3.1 Mastitis

**FIGURE 3:**
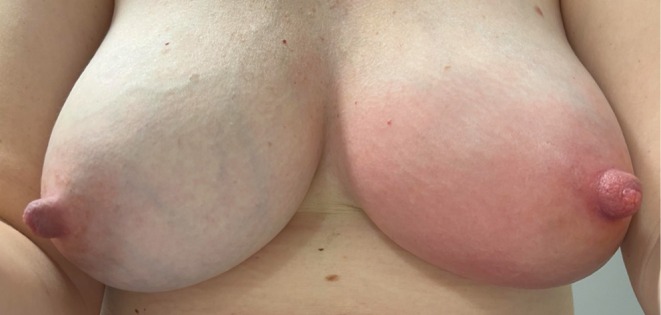
Mastitis. Image provided by Dr Annabel Stevenson.

**FIGURE 4:**
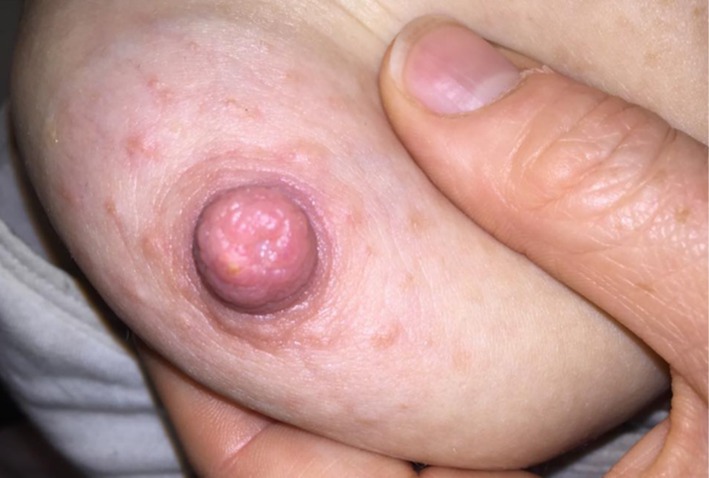
Milk bleb (7 o'clock on nipple). Image provided by Dr Annabel Stevenson.

Mastitis, the inflammation of lactiferous ducts, affects somewhere between 3% and 20% of lactating women, and its associated pain is a common reason for early cessation of breastfeeding [32]. Blocked lactiferous ducts result in stasis of breast milk, and clinically, the affected breast is tender to palpation, with variable erythema and engorgement of the affected duct, as seen in Figure 3. It usually presents unilaterally, most often in the first two to three weeks post‐partum and may be associated with fevers and myalgias. The nipple may exhibit a milk bleb (Figure 4), a white spot resulting from propagation of inflammatory cells, which become lodged at the opening of the duct. Secondary infection with *Staphylococcus* and *Streptococcus* species may occur in the area of stasis, eventually leading to abscess formation in some cases.

Risk factors for milk plugging include poor feeding technique, infrequent or irregular feeding, attempts to rapidly wean from feeding, oversupply of milk, and occlusion from clothing or bras. Discomfort from other breast pathologies, including breast dermatoses, also puts the mother at risk of milk stasis.

#### 2.3.2 Yeast Infection

**FIGURE 5:**
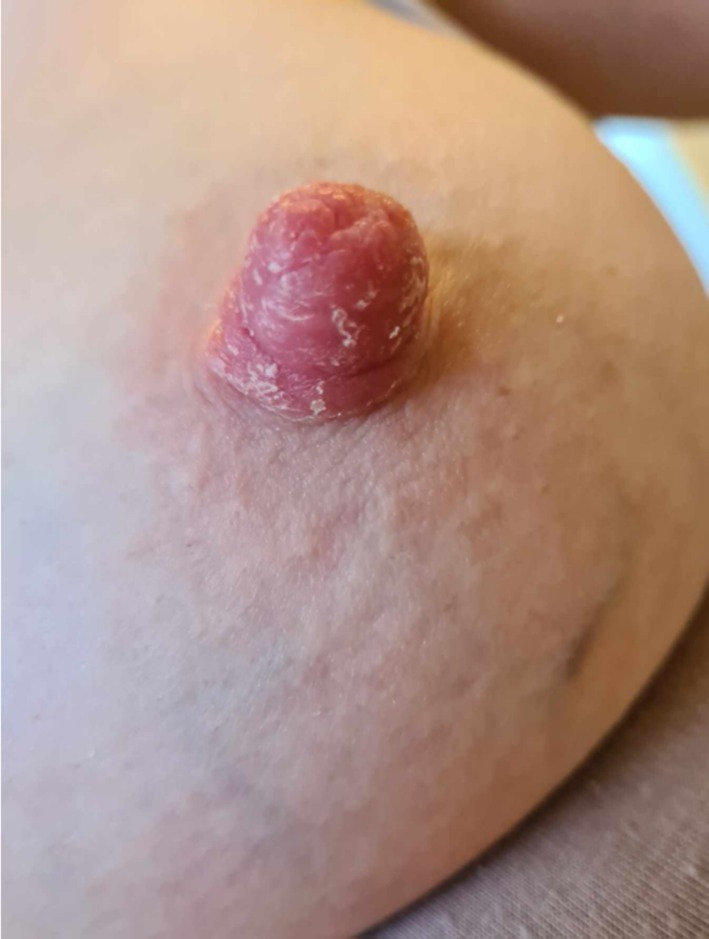
Candidiasis of the Nipple. Image provided by Dr Annabel Stevenson.

Yeast infections, most often *Candida spp*., of the breast and nipples, are commonly diagnosed during lactation. Usually bilateral in distribution, nipple candida is typically associated with a sharp shooting or burning pain, often out of proportion to physical examination findings. As seen in Figure 5, the infection may present with an erythematous, shiny nipple‐areola complex, often extending to the surrounding skin of the breast. Other clues to the diagnosis may include a personal history of candida infection, recent antibiotic use, and the onset of discomfort following previous pain‐free feeding. Concurrent or recent oral or nappy candida infection in the breastfeeding infant should be considered and may be an obvious sign [33, 34]. Unlike eczema, the skin immediately adjacent to the nipple is not spared [3].

The diagnosis of candida mastitis is usually made clinically, with several small studies showing that true candida mastitis may not correlate with the presence or absence of Candida growth on swabs [35, 36].

#### 2.3.3 Herpes Simplex Virus

Herpes Simplex Virus (HSV) infections most commonly affect the oral and anogenital regions, though herpetic mastitis has been infrequently reported. The condition is typically described as intensely painful or pruritic, with fine vesicles of the nipple‐areolar complex on an erythematous, swollen base, progressing to the development of “punched out” erosion [1, 37]. HSV infection of the breast most often presents unilaterally. Whilst sexual transmission and autoinoculation are possible, transmission from an infant to their breastfeeding mother is the most reported route of HSV infection of the breast [37].

#### 2.3.4 Herpes Zoster Virus

Herpes Zoster (HZV), or Shingles, results from reactivation of Varicella Zoster Virus during periods of immunosuppression. Following a prodrome of pain or itch, HZV presents as pruritic or tender vesicles, which may become haemorrhagic. The rash is circumscribed to a unilateral, dermatomal distribution, which naturally may include the breast or nipple, supplied by the T4 dermatome. In cases where the breast is affected, temporary cessation of breastfeeding is recommended. However, with contralateral or distant disease, cautious continued feeding is possible, ensuring the infant does not come in contact with the affected area [38].

### 2.4 Vascular Conditions

#### 2.4.1 Raynaud's Phenomenon of the Nipple

Exposure to cold temperatures, especially with sudden changes in temperature, may result in vasospasm of the blood vessels supplying the nipple, termed Raynaud's Phenomenon of the Nipple. This vasospasm results in ischaemia of the nipple and in turn, leads to pain and swelling. Whilst the condition may present in non‐lactating patients, exposure of the nipples during feeding may be the first time a nursing mother experiences the phenomenon [33]. The diagnosis is made clinically, based on nipple pain following exposure to cold, with or without the classic biphasic or triphasic colour changes described with Raynaud's Phenomenon. As with digital Raynaud's Phenomenon, smoking is an important risk factor [39].

Isolated Raynaud's Phenomenon of the digits has been reported in up to 20% of women of childbearing age. Whilst there has been no established correlation between the two conditions, in the largest published case series, 20 of the 22 patients with Raynaud's of the nipples had previously experienced symptoms elsewhere [39]. The published literature fails to comment on whether isolated Raynaud's Phenomenon of the Nipple warrants investigation for underlying connective tissue disease, though a complete review of systems would be prudent to investigate for symptoms of associated connective tissue disease [39, 40].

## 3 Investigation of Breast Dermatoses in the Lactating Woman

The diagnosis of breast dermatoses is largely based on history and physical examination. The history ought to clarify the timing and pattern of nipple pain and development of the dermatosis and should elucidate any risk factors or causative triggers outlined in Table 1. The treating doctor should focus not only on the health of the affected mother, but also on their breastfeeding infant as well. Physical examination should involve inspection of the affected area, as well as palpation to observe for underlying induration, fluctuance, or mass. The physical examination should equally include observation of breastfeeding or expression technique, examining the breast for resultant shape or colour changes, which may suggest traumatic feeding as a potential trigger.

**TABLE 1:** Summary of breast and nipple dermatoses in the lactating woman.

Name	Risk factors/Triggers	Method of diagnosis	Treatment
Irritant contact dermatitis	Triggers: Trauma (poor latch, ill‐fitting breast pump, frequent cleaning), moisture excess (saliva, sweat, emollients and topical therapy, occlusive clothing, nipple shields), chemical irritation Risk Factors: Congenital abnormalities (tongue tie, cleft lip, and palate)	Clinical diagnosis: Erythema, itch, pain, burning Acute: Oozing, crusting, erosions Chronic: Scaling, lichenification Areola is most affected (compared to nipple or adjacent skin)	General: Gently dab dry nipples after feeding Use emollient and non‐soap wash products Avoid padded bras Use nipple protection creams between feeding and exercise Avoid identifiable triggers (allergens, detergents, foods) Correction of poor latch, feeding technique, or breast pump use Treatment of congenital abnormalities (cleft, tongue tie) Topical: Gently wipe treatment from nipple before breastfeeding, apply immediately after feeding Regular emollient use First Line: Low‐to‐moderate potency topical corticosteroid ointment twice daily for two weeks or until symptoms resolve. Increase the potency of the steroid if required. Second line: Topical calcineurin inhibitors Systemic: UVB phototherapy (compliance may be difficult in postpartum period; note: PUVA contraindicated) Dupilumab (exercise caution given limited data) Oral corticosteroids (for severe, acute flares) Cyclosporin, Azathioprine
Allergic contact dermatitis	Triggers: Soaps, detergents, fabric softeners, fragrances, nipple shields/covers (rubber, silver, nickel, cobalt), topical remedies (lanolin, beeswax, chamomile, garlic, aloe vera, vitamin A and E), topical medications (antibiotics, antifungals), infant's oral medications or solid foods Risk Factors: History of atopic dermatitis	Clinical diagnosis: As for irritant contact dermatitis Nipple more likely to be affected than in irritant contact dermatitis, more likely to be geometric in outline Patch testing: Australian baseline series and cosmetic allergen series; additional testing targeted to history (topical medications, fragrances, textile dyes) – See Table 2	
Atopic dermatitis	Triggers: As for irritant contact dermatitis Risk Factors: Personal or family history of atopy (asthma, allergic rhinitis), smoking	Clinical diagnosis: As for irritant contact dermatitis May have associated patches of eczema in typically atopic areas (cubital and popliteal fossa)	
Psoriasis	Triggers: Koebnerisation from traumatic feeding or pumping, medications (beta‐blockers, lithium, antimalarials, NSAIDs, tetracyclines) Risk Factors: Personal history (post‐partum flares common), age (common in women of childbearing potential), obesity, hypertension, smoking, stress	Clinical diagnosis: Salmon‐coloured plaques with overlying scale, usually more well‐demarcated than eczema	General: Correction of poor latch, feeding technique or breast pump use Treatment of congenital abnormalities (cleft, tongue tie) Cessation of causative medication Topical: Gently wipe treatment from nipple prior to breast feeding, apply immediately after feed Regular emollient use First Line: Low‐moderate potency topical corticosteroid ointment twice daily for two weeks or until symptoms resolve Second line: High potency topical corticosteroid ointment twice daily for two weeks or until symptoms resolve Systemic/Physical: First line: UVB phototherapy (note: PUVA contraindicated) Second line: Cyclosporin, or Targeted Immunomodulators (i.e., “biologics”) (greatest evidence for safety in breastfeeding in older agents, see Table 4)
Mammary Paget's disease	Risk Factors: Personal or family history of breast cancer, increased age, obesity, radiation exposure, Caucasian	Punch Biopsy: >/=3 mm biopsy indicated in unilateral lesions unresponsive to treatment	Physical: Surgical resection in most cases (complete or partial mastectomy) Systemic: Adjuvant chemoradiotherapy in selected patients (may necessitate weaning)
Mastitis	Triggers: Milk stasis Risk Factors: Poor feeding technique, irregular feeding, rapidly weaning feeding, occlusion from clothing or bras, pain from other breast pathologies	Swab culture or breast milk culture: Assess for secondary bacterial infection	General: Continue regular breast feeding or general expression of breast milk Systemic (severe cases with systemic symptoms): Dicloxacillin or flucloxacillin 500 mg orally 6 hourly for 10 days (5 days may be sufficient in rapidly responsive cases)
Yeast Infection	Triggers: Infant oropharyngeal candidiasis Risk Factors: History of candida infection, recent antibiotic use, concurrent or recent infant nappy candidiasis, moisture excess (saliva, sweat, emollients and topical therapy, occlusive clothing or nipple shields)	Clinical diagnosis: Erythematous, shiny nipple‐areola complex, pain out of proportion to examination findings Culture swab: Positive culture does not necessarily correlate with symptoms	General: Thorough cleaning of clothing or equipment in contact with affect nipples or infant mucosa Topical (mother): Miconazole 2% cream twice daily to nipple and areola (wipe from nipple prior to breast feeding, apply immediately after feed) Topical (infant): Miconazole 2% oral gel or nystatin oral suspension 1mL qid orally for 7–14 days Systemic: Oral fluconazole 200 mg once, then 100mg daily for 7–10 days (for resistant cases)
Herpes Simplex	Triggers: Transmission from infant to mother most common route of infection	Viral PCR or culture: Swab taken from newly punctured vesicular lesion	General: Cessation of feeding from affected nipple or breast, pump and discard milk for comfort Systemic: Valaciclovir 500 mg, oral, BD for 5 days
Herpes Zoster	Triggers: Immunosuppression Risk Factors: Past infection with Varicella and/or Herpes Zoster	Viral PCR or culture: Swab taken from newly punctured vesicular lesion	General: Cessation of feeding from affected nipple or breast, pump and discard milk for comfort Systemic: Valaciclovir 1 g, oral, TDS for 7 days
Raynaud's phenomenon	Triggers: Cold exposure Risk factors: Smoking, caffeine, alcohol, vasoconstrictive medications	Clinical Diagnosis: Extreme pain, onset of symptoms with cold exposure, bi‐ or triphasic colour changes, occurrences without breastfeeding	General: Avoidance of cold exposure, warm compresses, avoidance of caffeine, nicotine and vasoconstrictive medications Systemic: Nifedipine Controlled‐Release 30–60 mg, oral, daily

**FIGURE 6:**
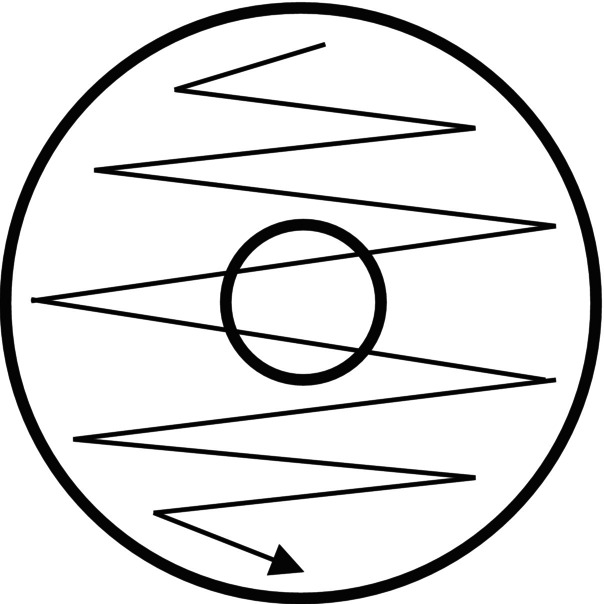
Culture swab technique. Original figure created by Dr Hamish Moore.

In cases of suspected infection, sampling for culture may be indicated. Where the skin is intact, a moist swab should sample the skin of the nipple‐areolar complex in a ten‐point zig‐zag fashion (Figure 6), before being placed in a culture medium. This may be required in suspected cases of yeast infection or mastitis, for example. In cases of suspected mastitis, additional milk culture may be necessary to determine the infecting organism. The nipple should be prepared using saline lavage and alcohol preparation, prior to discarding a few drops. 5–10 mL of milk should then be expressed manually to send for culture. Where there is an open wound, fissure, or vesicle, the moistened swab should be rotated in the affected area or at the site of a deroofed vesicle for five seconds before being sent for culture or PCR testing. Both swab and milk culture collection should occur under appropriately sterile conditions [2, 35, 127].

In cases of unilateral nipple dermatoses persisting for longer than three weeks or those not responding to treatment, Paget's disease of the nipple should be considered. Punch biopsy (at least 3mm) is recommended for patients with skin changes concerning for breast malignancy [128, 129]. In cases with an associated breast mass, the need for imaging with mammography or ultrasound, core biopsy, and referral to a breast clinic should be considered. The development of a milk fistula following punch biopsy has not been reported, though there is a theoretical risk. Regardless, lactation is not considered a contraindication to this necessary investigation [130, 131]. Emptying the breast prior to biopsy and applying pressure to the biopsy site when subsequently feeding can reduce the risk [131].

In cases of suspected contact allergy, patch testing should be performed. At present, there is no specific guideline for patch testing patients with breast and nipple dermatoses in Australia. Instead, the Skin Health Institute and the Contact Allergen Bank of Australia (CABA) recommend the use of the Australian Baseline Series (ABS) and the cosmetic allergen series, both of which are considered standard in this population. Based on our findings of reported cases of breast and nipple allergic contact dermatitis, we propose the following targeted “nipple series” (Table 2).

**TABLE 2:** Reported allergens causing allergic contact dermatitis of breast and/or nipples.

Allergen	Reported source of exposure
Nickel sulfate hexahydrate^a^	Nickel in mobile phone
Disperse blue 106 fabric dye (Textile dye mix^a^)	Fabric dye used in lining of blue dress
Methylisothiazolinone^a^	Ingredient in baby wipes
Propylene glycol^a^	Component of bra padding
Chamomile (Compositae Mix II^a^)	Chamomile used as home remedy for topical analgesia
Propolis^a^	Beeswax used as home remedy for topical analgesia
Diallyl Disulfide	Garlic poultice used on nipples as home remedy for topical analgesia
Aloe Vera	Aloe vera used as home remedy for topical analgesia
Lanolin^a^	Used as home remedy for topical analgesia
Miconazole	Ointment used to treat nipple candidiasis
Sodium metabisulphite^a^	Ingredient in Trimovate cream

^a^ Included in Australian Baseline Series (ABS).

## 4 Management of Dermatoses in the Lactating Woman

Treatment of dermatoses in a woman who is lactating requires special consideration of the safety of the infant. Infants may be exposed to medications via breastmilk, or in the case of topical medications, through direct contact with the product. Uniquely with breast and nipple dermatoses, topical products are also at risk of ingestion. The next section will examine the known literature surrounding the safety of medications commonly used in dermatology.

### 4.1 Topical Medications

#### 4.1.1 General Advice

Topical medications are commonly seen in dermatology. Breastfeeding infants may be at undue risk of exposure to their mother's medication through several mechanisms. Firstly, these medications may be absorbed systemically and transferred via the breast milk. Secondly, the infant may come in direct contact with the treated area, exposing their own skin to the treatment and its side effects. Finally, the infant is at risk of ingestion of products applied to the nipple and breast area.

The safety profile of topical medications during breastfeeding is outlined in Table 3. Whilst each medication is unique, when used in the treatment of breast and nipple dermatoses, some general recommendations apply. Patients should apply their medication immediately after each feed, allowing for maximal absorption prior to the next feed. Before feeding again, it is recommended to gently wipe the treated area with a warm towel to reduce infant oral exposure. Avoiding traumatic cleaning and ensuring the breast is dry will reduce exacerbating some dermatoses. Lactating mothers should also seek to use the lowest concentration of medications for the shortest time possible to further reduce exposure.

**TABLE 3:** Summary of medication safety during lactation and breastfeeding—topical [27, 28, 29, 41, 42, 43, 44, 45, 46, 47].

Topical	Mechanism of action	Safety in breastfeeding		Reported risks in infant
		General use	Use on Nipple/Areola	
Topical steroids				
Hydrocortisone 1%	Corticosteroid	Safe	Safe	A case report of a mother using topical isoflupredone acetate (corticosteroid) on the nipple resulted in QT prolongation, hypertension cushingoid appearance and electrolyte abnormalities in her two‐month‐old infant [132]
Methylprednisolone aceponate 0.1%	Corticosteroid (predominantly glucocorticoid)	Safe	Safe	
Triamcinolone acetonide 0.02%	Glucocorticoid	Safe	Safe	
Betamethasone valerate 0.02%	Glucocorticoid	Safe	Benefit must outweigh risk Use of ultra‐ and high potency steroids on the nipple is rarely indicated	
Mometasone furoate 0.1%	Corticosteroid (predominantly glucocorticoid)	Safe		
Betamethasone diproprionate and betamethasone diproprionate OV 0.05%	Glucocorticoid	Safe		A mother treated with oral prednisolone, cetirizine, topical betamethasone and topical clobetasol reported no effects in her breastfeeding newborn [71]
Clobetasol proprionate 0.05%	Corticosteroid (predominantly glucocorticoid)	Safe		
Calcineurin Inhibitors				
Pimecrolimus	Calcineurin inhibitors Disrupt transcription of IL‐2 in T‐lymphocytes	Safe	Safe (wipe off prior to feeding)	No reports of use during breastfeeding
Tacrolimus				
Crisabarole	PDE4 Inhibitor	Benefit must outweigh risk No published data Manufacturers suggest safe for general use		No reports of use during breastfeeding
Calcipotriene	Vitamin D Analogue	Safe	Avoid	No reports of use during breastfeeding
Diphenylcyclopropenone (DPCP)	Topical immunomodulator reducing autoimmune attack on hair follicles	Avoid	Avoid	No reports of use during breastfeeding
Dithranol/Anthralin	Reduced mitotic activity of hyperactive epidermis	Benefit must outweigh risk	Avoid	No reports of use during breastfeeding
Retinoids				
Tazarotene	Binds RAR receptors, decreasing keratinocyte proliferation and differentiation	Avoid	Avoid	No reports of use during breastfeeding
Tretinoin	Vitamin A derivative, binds RAR and RXR receptors reducing keratinocyte cohesiveness and follicular occlusion	Safe Minimal systemic absorption, likely safe Avoid contact with infant skin	Avoid	No reports of use during breastfeeding
Adapalene	Binds RAR receptors reducing keratinocyte cohesiveness and follicular occlusion	Safe Minimal systemic absorption, likely safe Avoid contact with infant skin	Avoid	No reports of use during breastfeeding
Benzoyl peroxide	Bacteriostatic (against C. acnes) and comedolytic	Benefit must outweigh risk Minimal systemic absorption, likely safe Avoid contact with infant skin	Avoid	No reports of use during breastfeeding
Azelaic acid	Inhibits growth of C. acnes and inhibits keratinocyte differentiation and proliferation	Safe Minimal systemic absorption Normal component of breastmilk Avoid contact with infant skin	Avoid	No reports of use during breastfeeding
Salicylic acid	Beta‐hydroxy acid, keratolytic and mild antibacterial and antifungal	Safe Minimal absorption and secretion in breastmilk Use lowest dose (< 3%) for shortest time possible	Avoid Risk of Reye's Syndrome	No reports of use during breastfeeding
Minoxidil	Increases duration of anagen and enlarges miniaturised hair follicles	Benefit must outweigh risk	Avoid	A case report of facial hypertrichosis in the breastfed infant of a mother using topical minoxidil [133] A case report of a 7‐year‐old girl who accidentally ingested topical minoxidil developed significant hypotension
Coal tar	Poorly understood; suppression of DNA synthesis and thus keratinocyte proliferation	Benefit must outweigh risk Detectable in infant's urine in one case report [134]	Avoid	Single case report, 3‐month‐old and mother using tar on all areas but face and breasts; tar metabolites detectable in urine of infant, despite being undetectable in breastmilk, suggesting infant‐skin contact [134]
Antibiotics				
Erythromycin	Macrolide antibiotic; inhibit bacterial protein synthesis by binding to the 50S ribosomal subunit	Safe	Benefit must outweigh risk Risk of diarrhoea	No reports of use during breastfeeding
Clindamycin	Lincosamide antibiotic; inhibit bacterial protein synthesis by binding to the 50S ribosomal subunit	Safe	Benefit must outweigh risk Risk of diarrhoea	No reports of use during breastfeeding
Mupirocin	Monoxycarbolic acid; inhibits bacterial protein synthesis in gram‐positive and some gram‐negative aerobes	Safe	Safe	A breastfeeding mother was treated with IV teicoplanin, ceftriaxone, topical mupirocin (not on nipples) with no effects in infant [135]
Fusidic acid	Binds EF‐G, resulting in impaired bacterial protein synthesis	Safe	Safe	17 patients in one randomised, unblinded study of mothers at risk of mastitis used fusidic acid on their nipples; infant outcomes were not reported [136]
Metronidazole	Nitroimidazole antibiotic; inhibits nucleic acid synthesis disrupting microbial cellular DNA	Avoid	Avoid	No reports of topical use during breastfeeding Reports of candida infection and diarrhoea with systemic use [137, 138, 139, 140]
Antifungals				
Clotrimazole	Impair synthesis of ergosterol in fungal cell membranes leading to their breakdown	Safe	Safe	No reports of use during breastfeeding
Miconazole		Safe	Safe	No reports of use during breastfeeding
Ketoconazole		Safe	Avoid Risk of ingestion and safer alternatives available	No reports of topical use in breastfed infants A mother using oral ketoconazole noticed no effects in her breastfed infant [141]
Antiparasitic				
Ivermectin	Binds glutamate‐gated chloride ion channels, acting as a GABA‐antagonist, causing parasitic paralysis and death	Safe	Avoid	No reports of topical use during breastfeeding
Excipients/Bases				
Mineral oil	Occlusive effect, minimising transepidermal fluid losses	Safe	Avoid	One study suggests direct application to the nipple may result in ingestion of up to ten times the acceptable daily dose in the breastfed newborn [142]
Paraffin wax/oil				
Intralesional agents				
Lignocaine	Sodium channel blocker, thus inhibiting depolarization and impulse conduction in peripheral nerves	Safe	Benefit must outweigh risk	No reports of use during breastfeeding
Triamcinolone	Glucocorticoid	Safe	Benefit must outweigh risk	A temporary but significant decrease in milk production noted after injection of triamcinolone into breast to treat idiopathic granulomatous mastitis; triamcinolone not detected in breast milk [143]
Botulinum Toxin A (Botox) and AbobotulinumtoxinA (Dysport)	Blocks release of acetylcholine resulting in paralysis	Benefit must outweigh risk Avoid administration for cosmetic causes	Benefit must outweigh risk Avoid administration for cosmetic causes	In a breastfeeding mother suffering from botulism, no toxin was detectable in the breast milk nor in the infant's serum [144]

*Note:* Significance of Green, Yellow, Red indicates as follows: Green = Medication is safe to use during breastfeeding. Yellow = Benefit of use must outweigh risks during breastfeeding. Red = Avoid use during breastfeeding.

#### 4.1.2 Preparation of Medication

With regards to topical medications, an area inconsistently reported on in the literature is the safety of ointments containing mineral oils on the nipples and areolae of breastfeeding mothers. Whilst reporting of medication safety is often focused on the active ingredient, information on the additives of products, which are at risk of ingestion by the breastfeeding infant, is frequently omitted, despite clearly being relevant in this group.

Some guidelines have advocated for the use of ointments over water‐miscible cream or gel products, claiming that because of their occlusive properties, ointments are better absorbed with topical use [2, 3]. Whilst this may be the case, other experts suggest avoiding the use of ointments on the nipples of breastfeeding patients wherever possible [29, 41], after a frequently cited study of breastfeeding subjects suggested that by using paraffin oil on a mother's nipples, an infant could ingest up to ten times the recommended daily dose of mineral hydrocarbons [142]. Notably, no such concern has been demonstrated for microcrystalline waxes, which, owing to their higher molecular weight, are considered safe for ingestion. Lanolin, which is not a hydrocarbon but is rather composed of fatty acid esters, is also not of concern.

Given the lack of definitive data, dermatologists should exercise caution when prescribing mineral oil‐, paraffin‐ and wax‐containing products for use on the breast area of lactating mothers. Whilst prolonged oral ingestion of some ointments may be harmful, with sparing use and judicious cleaning of the nipple prior to breastfeeding, these products are likely to be safe for short‐term treatment. One approach may be to use a high‐potency ointment formulation for a short period, before transitioning to a water‐based product when the disease is better‐controlled.

### 4.2 Systemic Medications

#### 4.2.1 General Advice

Systemic medications, typically taken orally or subcutaneously, may be transferred to the breastfeeding infant via the breast milk. Many are safe for use during lactation, as summarised in Table 4.

**TABLE 4:** Summary of medication safety during lactation and breastfeeding—systemic [27, 28, 29, 41, 42, 43, 44, 45, 46, 47].

Systemic agents	Mechanism of action	Safety in breastfeeding	Reported risks in infant
Oral antibiotics			
Doxycycline	Tetracycline antibiotic; inhibit bacterial protein synthesis by binding to the 30S ribosomal subunit	Safe for short periods (< 21 days) Minimal transfer in breast milk Poor absorption due to calcium binding Avoid prolonged or repeat use (> 21 days) due to risk of dental staining	None reported
Minocycline	Tetracycline antibiotic; inhibit bacterial protein synthesis by binding to the 30S ribosomal subunit	Safe for short periods (7–10 days) Minimal transfer in breast milk Poor absorption due to calcium binding Avoid prolonged or repeat use (> 21 days) due to risk of dental staining	Two reported cases of black galactorrhoea with long‐term oral minocycline use [48, 49]
Erythromycin	Macrolide antibiotic; inhibit bacterial protein synthesis by binding to the 50S ribosomal subunit	Safe Risk of diarrhoea and candidiasis in infant	Two studies suggested a possible association between hypertrophic pyloric stenosis and use during breastfeeding [50, 51] Two meta‐analyses failed to demonstrate a causative relationship [52, 53]
Clindamycin	Lincosamide antibiotic; inhibit bacterial protein synthesis by binding to the 50S ribosomal subunit	Safe Risk of diarrhoea and candidiasis in infant	Single case report of newborn developing bloody stools after mother administered IV Clindamycin and Gentamicin [54]
Cephalexin	Cephalosporin antibiotic; bind to penicillin‐binding proteins, interfering with bacterial cell wall peptidoglycan synthesis	Safe Minimal transfer in breastmilk Risk of diarrhoea in infant	In 7 mothers taking cefalexin, 2 breastfed infants developed diarrhoea [55] In 11 mothers taking cefalexin, 1 breastfed infant developed diarrhoea [56] In a mother receiving IV cefalothin then oral cefalexin, the breastfed infant developed diarrhoea [57] A 4‐month‐old infant developed toxic epidermal necrolysis follow IV Cefazolin administration; prior sensitisation to cefalexin through breast milk was thought to be a trigger [58]
Flucloxacillin	Penicillin antibiotic; bind to penicillin‐binding proteins, interfering with bacterial cell wall peptidoglycan synthesis	Safe Minimal transfer in breastmilk Risk of diarrhoea in infant	No reports of use during breastfeeding
Trimethoprim/Sulfamethoxazole (Bactrim)	Bacteriostatic through competitive inhibition of bacterial folate production	Safe in healthy, full‐term infant Avoid in ill or pre‐term infants, especially those with hyperbilirubinemia or G6PD deficiency	A systematic review found no adverse effects on the use of trimethoprim/sulfamethoxazole during breastfeeding, including no neonatal kernicterus [59] In a prospective study of 12 mothers taking trimethoprim/sulfamethoxazole during breastfeeding, two mothers reported poor feeding in their newborn [60]
Hormonal treatments			
Combined Oral Contraceptive Pill	Suppression of androgens, decreasing sebum production	Benefits must outweigh risk Avoid for 6 weeks post‐partum due to risk of VTE in mother May reduce milk production	Multiple case reports and case series of transient gynaecomastia in breastfed infants of mothers taking OCP; resolution of symptoms with cessation of use [61, 62, 63, 64] One case report of an infant developing folate deficiency possibly caused by maternal OCP use [65] At 8 year follow‐up, no difference in growth and development of 48 breastfed children whose mothers started taking OCP at two months post‐partum [66]
Spironolactone	Suppression of androgens, decreasing sebum production	Safe	No adverse effects in a case report of a 17‐day‐old infant [67]
Retinoids			
Acitretin	Binds RAR and RXR receptors reducing keratinocyte proliferation	Avoid	No reports of use during breastfeeding
Isotretinoin	Modulates cell proliferation and differentiation, including decreased sebum production	Avoid	No reports of use during breastfeeding
Photosensitising agents			
Psoralens (Methoxsalen)	Once activated by UV light, bind to DNA and inhibit DNA synthesis	Avoid for 24 h after oral dose	No reports of use during breastfeeding
Methyl aminolevulinate (Topical)	Photosensitising agent; activated by light to produce reactive singlet oxygen, destroying tumour cells	Avoid Discontinue breastfeeding for 48 h after application	No reports of use during breastfeeding
Antivirals			
Aciclovir	Guanine analogues Inhibit viral DNA polymerase and DNA synthesis	Safe	None reported [46, 68]
Valaciclovir		Safe	None reported [46, 69]
Famciclovir		Benefit must outweigh risk	Secreted at higher concentration than Acyclovir/Valaciclovir and no reports of safe use, so considered second‐line option [46]
Antifungals			
Fluconazole	Impair synthesis of ergosterol in fungal cell membranes leading to their breakdown	Safe	In a study of 96 mothers taking oral fluconazole, 7 breastfed infants developed possible minor side effects (flushed cheeks, GI upset) [70]
Itraconazole		Benefit must outweigh risk Use of alternate agents is recommended first line For long treatment courses, monitor LFTs	No reports of use during breastfeeding
Terbinafine	Inhibits squalene epoxidase, thereby inhibiting fungal ergosterol synthesis leading to cell death	Benefit must outweigh risk Avoid in infants < 2mo Monitor infant for signs of liver toxicity (i.e., jaundice), especially younger breastfed infants	No reports of use during breastfeeding
Griseofulvin	Disrupts fungal cell microtubule formation	Avoid Tumorigenic potential	No reports of use during breastfeeding
Antiparasitic			
Ivermectin	Binds glutamate‐gated chloride ion channels, acting as a GABA‐antagonist, causing parasitic paralysis and death	Safe	No reports of use during breastfeeding
Antihistamines			
Cetirizine	Second‐generation antihistamines Bind H1 receptors, thus inhibiting histamine activity	Safe Monitor for signs of excessive irritability or drowsiness with prolonged use High dose antihistamines may reduce prolactin secretion in early postpartum women, which may affect lactation establishment	Multiple case reports and series with no adverse effects on the breastfed infant [55, 71, 72, 73] In one study of 31 women taking cetirizine, 39% noticed minor side effects (fever, rash, sedation, constipation, poor feeding), most of which were attributed to other causes [74]
Fexofenadine			In one study, three out of 25 women taking fexofenadine reported irritability in their breastfeeding infant [55]
Loratidine			In one study, two out of 51 women taking loratadine reported sedation in their breastfeeding infant [75]
Desloratidine			No reports of use during breastfeeding
Promethazine	First‐generation antihistamine	Benefit must outweigh risk Risk of sedation in the infant Risk of interference with lactation establishment	No reports of use during breastfeeding
Immunosuppressive/immunomodulatory agents			
Methotrexate	Folic acid antagonist with both antineoplastic and immunomodulatory properties	Antineoplastic doses: Avoid Immunomodulatory doses: Benefit must outweigh risk Withhold breastfeeding for at least 24 h after weekly, low dose Monitor infant liver function tests and blood counts	A mother taking 25mg weekly methotrexate breastfed her 151‐day‐old infant for 9 months with no adverse effects [76] Three breastfeeding mothers inadvertently received toxic doses of methotrexate, with no observable adverse effects in their infants [77]
Azathioprine	Metabolised to thioguanine nucleotides which interfere with purine synthesis, impairing lymphocyte proliferation, cellular immunity and antibody responses	Safe Monitor of full blood count and liver function tests in infant Avoid breastfeeding for 4 h after dosing	Multiple case reports and case series with no reported adverse effects on breastfeeding infants [78, 79, 80, 81, 82, 83, 84, 85, 86] One of six infants breastfed by mothers taking azathioprine developed a “low blood count” [87] One of ten infants breastfed by mothers taking azathioprine developed asymptomatic mild neutropenia after 28 days [88] Increased rates of childhood infection requiring hospital admission [89] 21 mothers taking allupurinol and a thiopurine (azathioprine or mercaptopurine) breastfed their infants; two infants died, one of issues related to prematurity and one of SIDS; the authors did not believe this was related to their medication 59 infants exposed to azathioprine during pregnancy and breastfeeding were reported to have higher rates of infection compared to 114 infants who were not [89]
Mycophenolate Mofetil	Inhibits inosine monophosphate dehydrogenase, selectively suppressing lymphocyte proliferation and antibody formation	Avoid Secreted in breastmilk Potential for serious adverse events including immunosuppressi‐on and lymphoma	No adverse effects were observed in the children of two breastfeeding women taking mycophenolate [86] Six mothers breastfed seven infants without adverse outcomes whilst taking mycophenolate [84]
Apremilast	PDE‐4 inhibitor, reducing pro‐inflammatory cytokines	Avoid No human data Manufacturer contraindicates use	No reports of use during breastfeeding
Deucravacitinib	Tyrosine Kinase 2 inhibitor	Avoid No human data	No reports of use during breastfeeding
Cyclosporin	Calcineurin inhibitor; disrupt transcription of IL‐2 in T‐lymphocytes	Safe Monitor infant drug levels	Multiple case reports and case series with no reported adverse effects on breastfeeding infants [17, 79, 84, 88, 90, 91, 92]
Prednisolone	Systemic corticosteroid (predominantly glucocorticoid)	Safe Avoid breastfeeding for 4 h after high doses (i.e., > 80 mg) Large doses may temporarily decrease lactation High doses for a prolonged period could impact on infant growth and development	Multiple case reports and case series with no reported adverse effects on breastfeeding infants [55, 71, 88, 93, 94, 95]
Dapsone	Antibacterial through inhibition of folic acid synthesis Anti‐inflammatory through inhibition of myeloperoxidase in polymorphonucleocytes	Benefit must outweigh risk Monitor infant for signs of haemolysis and jaundice Consider alternate agent in pre‐term/newborn infants and infants with G6PD deficiency	The 41‐day‐old infant of a breastfeeding mother taking 50mg dapsone daily developed haemolytic anaemia [96]
Hydroxychloroquine	Anti‐inflammatory through increasing lysosomal pH Antiproliferative and immunomodulatory through decreasing lymphocyte proliferation and natural killer cell activity	Safe	Multiple case reports and case series with no reported adverse effects on breastfeeding infants [89, 97, 98, 99, 100]
Upadacitinib	JAK‐1 inhibitor	Avoid Manufacturer recommends cessation for six days prior to feeding	No reports of use during breastfeeding
Tofacitinib	JAK‐1,2 and 3 inhibitor	Avoid Manufacturer recommends cessation for 18 days (immediate release) or 36 days (sustained release) prior to breastfeeding	No reports of use during breastfeeding
Baricitinib	JAK‐1 and 2 inhibitor	Avoid Manufacturer recommends cessation for four days prior to breastfeeding	No reports of use during breastfeeding
Intravenous Immunoglobulin Therapy	Immunoglobulins from human plasma having undergone viral inactivation	Safe Secreted into breastmilk; avoid live vaccination for three weeks before or six weeks after administration	In one case report [101], and one infant in a case series of mothers taking IVIg [102], the breastfed infants developed a transient rash In multiple case series, no adverse effects were noted in the breastfed infants [93, 103, 104]
Calcium Channel Blockers			
Nifedipine	Calcium channel blocker; act on L‐type calcium channels to block inward current of calcium, resulting in reduced vascular resistance	Safe	None reported [105, 106, 107]
Other			
Minoxidil	Increases duration of anagen and enlarges miniaturised hair follicles	Benefit must outweigh risk Caution with large maternal doses and newborn infants	One case report with no adverse effects in the breastfed infant [108]
Bicalutamide	Competitively inhibits the binding of androgens at androgen receptors	Avoid	No reports of use during breastfeeding
Biologic Agents			
Etanercept	TNF‐alpha inhibitor	Safe	Several case series with no adverse effects reported [109, 110, 111] A self‐resolving, mild rash was reported in one infant [112]
Adalimumab	TNF‐alpha inhibitor	Safe	Multiple case reports or series with no adverse effects reported [110, 113, 114, 115, 116, 117, 118]
Infliximab	TNF‐alpha inhibitor	Safe	Multiple case reports and case series with no adverse effects reported One of two twins breastfed by a mother taking Infliximab was hospitalised at 18 months with severe asthmatic bronchitis; at 24 months both twins were developing normally [119]
Certolizumab Pegol	TNF‐alpha inhibitor	Safe	Several case reports and case series with no adverse effects reported [110, 118, 120, 121, 122]
Ustekinumab	IL‐12 and IL‐23 inhibitor	Benefits must outweigh risk Likely safe but limited data	Multiple case reports and case series with no adverse effects reported
Guselkumab	IL‐23p19 inhibitor	Benefits must outweigh risk Likely safe but limited data	No reports of use during breastfeeding
Tildrakizumab	IL‐23p19 inhibitor	Benefits must outweigh risk Likely safe but limited data	No reports of use during breastfeeding
Risankizumab	IL‐23p19 inhibitor	Benefits must outweigh risk Likely safe but limited data	No reports of use during breastfeeding
Secukinumab	IL‐17A inhibitor	Benefits must outweigh risk Likely safe but limited data	No reports of use during breastfeeding
Ixekizumab	IL‐17A inhibitor	Benefits must outweigh risk Likely safe but limited data	No reports of use during breastfeeding
Bimekizumab	IL‐17A, IL‐17F, IL‐17AF inhibitor	Benefits must outweigh risk Likely safe but limited data	No reports of use during breastfeeding
Dupilumab	IL‐4 and IL‐13 inhibitor	Benefits must outweigh risk Likely safe but limited data	Two case reports and one case series demonstrated no adverse effects on the infant during breastfeeding [123, 124, 125]
Omalizumab	IgE inhibitor	Safe	Several case series and one large‐scale pregnancy registry observed no adverse effects in hundreds of breastfed infants [126]

*Note:* Significance of Green, Yellow, Red indicates as follows: Green = Medication is safe to use during breastfeeding. Yellow = Benefit of use must outweigh risks during breastfeeding. Red = Avoid use during breastfeeding.

There are numerous factors which may influence the excretion of a medication in the breastmilk. To some extent, most medications diffuse passively into breastmilk. As a result, feeding at the time of peak maternal plasma concentration will result in a larger dose to the infant. This also means that medications highly bound to maternal plasma proteins are less likely to be excreted. The size of the drug molecule plays another important role, with larger molecules, such as the newer biological medications, being less likely to be excreted [27, 28, 29]. Owing to the acidity of breastmilk compared to the serum, alkaline medications diffuse in higher concentration into breastmilk [145].

Additional factors unique to the infant also play an important role. The frequency and volume of breastfeeding will result in a change in the total dose received. The age of the infant is important, as increasing age correlates not only with increased child weight, but also with a significantly improved metabolism. Many of the medication‐related adverse outcomes reported have been seen in newborn or premature infants. The bioavailability of medications also plays a role, with some medications destroyed or poorly absorbed in the infant's GI tract after ingestion [145].

#### 4.2.2 Targeted Immunomodulators and Live Vaccinations in Breastfed Children

A consideration in patients prescribed targeted immunomodulator “biologic” therapy is the administration of vaccinations. Live‐attenuated vaccines are contraindicated in these patients, given the risk of disseminated infection. In general, this recommendation also applies to newborns exposed to biologic medications in utero. Whilst outside of the scope of this paper, most of these medications cross the placenta, with detectable levels of these drugs in a newborn's serum. In a single case report, a child exposed to Infliximab in utero died following administration of the BCG vaccine [146]; and as such, delaying the administration of live vaccines until 12 months of age is recommended. On the Australian immunisation schedule, the rotavirus vaccine is the only recommended live‐attenuated vaccine before 12 months (due at 2 and 4 months of age). The measles‐mumps‐rubella vaccine is due at 12 months and again at 18 months, when varicella is also contained in the live vaccine.

In cases where the infant was not exposed in utero, but where the mother has recommenced biologic treatment whilst breastfeeding, the impact on a child's immunisation schedule is less straightforward. Most biologics are excreted in the breastmilk at insignificant doses, and there is no conclusive evidence demonstrating that this exposure results in immunocompromise. Current guidelines on the use of these medications in the post‐partum period frequently fail to comment on the safety of infant vaccinations, defaulting to recommendations for patients exposed in utero. There are no case reports of the administration of live‐attenuated vaccinations in this population, successful or otherwise.

Ultimately, a nuanced and individualised approach is required. The treating dermatologist and their patient ought to take into consideration the mother's dermatosis, the risk of infection in an unvaccinated newborn, and the known benefits of continuing breastfeeding. Input from a paediatrician and monitoring of newborn drug levels are recommended if the decision is made to proceed with vaccination.

## 5 Conclusion

Breast and nipple dermatoses are not uncommon during lactation and breastfeeding. Their impact on a breastfeeding mother may significantly hinder the breastfeeding experience, ultimately leading to early cessation of breastfeeding. Dermatologists ought to have a thorough understanding of breast and nipple dermatoses in this population, including the impact of treatment on the nursing infant. Owing to their exclusion from clinical trials, safety data in this population is limited, and so a nuanced, patient‐focused approach is pivotal.

## Conflicts of Interest

The authors declare no conflicts of interest.

## Data Availability

Data sharing not applicable to this article as no datasets were generated or analysed during the current study.
